# Exploring the Clinical Applications of Lemongrass Essential Oil: A Scoping Review

**DOI:** 10.3390/ph17020159

**Published:** 2024-01-25

**Authors:** Ikhwan Yuda Kusuma, Muhammad Iqbal Perdana, Csaba Vágvölgyi, Dezső Csupor, Miklós Takó

**Affiliations:** 1 Pharmacy Study Program, Faculty of Health, Harapan Bangsa University, Purwokerto 53182, Indonesia; ikhwanyudakusuma@uhb.ac.id; 2 Institute of Clinical Pharmacy, University of Szeged, Szikra utca 8, H-6725 Szeged, Hungary; csupor.dezso@szte.hu; 3 Department of Microbiology, Faculty of Science and Informatics, University of Szeged, Közép fasor 52, H-6726 Szeged, Hungary; csaba@bio.u-szeged.hu

**Keywords:** lemongrass, medicinal plant, essential oil, clinical application, therapy, databases of research publications, systematic literature search, PRISMA guidelines

## Abstract

Lemongrass is a medicinal plant that produces essential oil with a variety of therapeutic properties. Although lemongrass essential oil (LGEO) is promising in clinical applications, the existing knowledge on the efficacy and safety of LGEO remains limited. This scoping review aimed to identify, summarize, and synthesize existing literature related to the clinical applications of LGEO to provide an overview of its potential therapeutic benefits for patients. Three databases (PubMed, Web of Science, Scopus) were used following the PRISMA-ScR guidelines to find articles published between 1 January 2013, and 1 November 2022. A total of 671 records were identified and 8 articles were included in this scoping review. The majority of patients received oromucosal and topical treatment. The results of the studies suggest that LGEO might be a useful tool in the treatment of periodontitis, gingivitis and oral malodour, with similar efficacy to chlorhexidine (anti-gingivitis effect) and doxycycline (periodontitis). Additionally, LGEO has the potential for treating pityriasis versicolor and preventing skin aging and may have anti-dandruff effects. These findings not only underscore the diverse clinical potential of LGEO but also emphasize its comparable efficacy to established treatments. Further research is imperative to comprehensively evaluate LGEO’s effectiveness, safety, mechanisms of action, potential interactions with other medications, and its long-term tolerability across diverse populations.

## 1. Introduction

Due to their affordability and accessibility, medicinal plants, natural products, and herbal diets have been used in traditional, complementary, and alternative medicine since ancient times [1]. Traditionally, natural products have been the mainstay for treating illnesses and wounds. Recent advances in molecular biology, genome mining, analytical techniques, and cultivation systems also allow natural products to remain promising materials for future drug developments [2].

The genus *Cymbopogon* comprises of around 180 species, subspecies, varieties, and subvarieties and it is a member of the Poaceae family [3,4]. *Cymbopogon* species are native to the tropical regions of Asia, Africa and South America. The members of this taxon yield different essential oils, namely lemongrass oil (obtained from *Cymbopogon citratus* (D.C.) Stapf., *Cymbopogon flexuosus* (Steud.) Wats., and *Cymbopogon pendulus* (Nees ex Steud.) Wats.), palmarosa or ginger grass oil (obtained from *Cymbopogon martinii*) and citronella oil (obtained from *Cymbopogon nardus* and *Cymbopogon winterianus*) [4]. The lemongrass essential oil (LGEO) can be further classified as West Indian lemongrass oil (*C. citratus*), East Indian lemongrass oil (*C. flexuousus*) and North Indian lemongrass oil (*C. pendulus*). However, *C. citratus*, *C. flexuosus*, and *C. pendulus* produce oil of almost identical chemical composition, hence the use of the collective term lemongrass oil is an acceptable compromise [3]. LGEO can be obtained through various extractions methods, e.g., hydrodistillation and solvent-free microwave-based extraction [5]. Other effective extraction approaches can be the solvent extraction, supercritical fluid extraction, and solar energy extraction methods [6].

**Figure 1:**
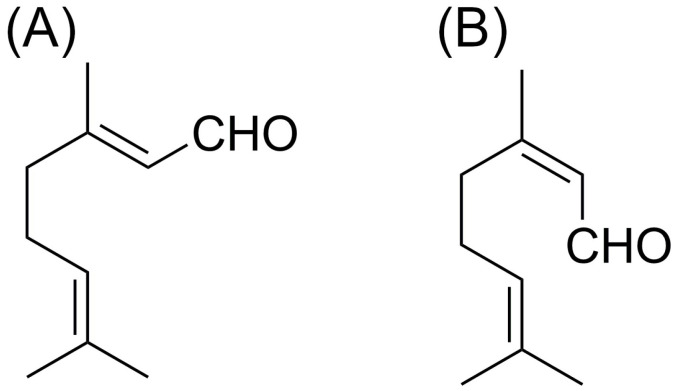
Chemical structures of the geometric isomers geranial (**A**) and neral (**B**).

The essential oils of the *Cymbopogon* species mainly comprises of mono- and sesquiterpenoids. The primary component of LGEO is the monoterpene citral, which has two distinct geometric isomers. Geranial (*trans*-citral; α-citral or citral A) is the name of the *E*-isomer, whereas neral (*cis*-citral; β-citral or citral B) is the name of the *Z*-isomer (Figure 1). However, the compositions of different lemongrass oils show some differences, due to extrinsic (growing and climatic conditions) and intrinsic (genetic) factors. The essential oil of *C. flexuosus* is characterized by the terpenoids geranial (40–50%) [3], neral (30–35%) [3], borneol (0–2%) [3], citronellal (0.4–8%) [3], citronellol (0.4–4.6%) [3], citronellyl acetate (1.2–3.6%) [3], geraniol (1.7–40%) [3], methyl eugenol (20%) [3], myrcene (0.1–14.2%) [3], geranyl acetate (2–5%) [3], and limonene (2.4–3.7%) [3]. In the *C. citratus* oils, geranial (10–48%) [3], neral (3–43%) [3], myrecene (12–15%) [3], borneol (5%) [3], geraniol (2.6–40%) [3], geranyl acetate (0.1–3%) [3], linalool (1.2–3.4%) [3], and nerol (0.8–4.5%) [3] are the main components. The essential oil of *C. pendulus* contains geranial (30–50%) [3], neral (20–35%) [3], geranyl acetate (3–5%) [3], β-caryophyllene (2.1%) [3], elemol (2.2%) [3], geraniol (2–6%) [3], and linalool (3%) [3] as main components. The essential oil of *C. flexuosus* contains the highest amount of citral [7]. Owing to its lower citral content, *C. citratus* oil is regarded as less valuable; however, when East Indian lemongrass oil became scarce following World War II, this oil gained significance. *C. pendulus* also provides lemongrass oil, but its cultivation is limited compared to the other two species. In contrast to *C. flexuosus* and *C. pendulus*, which are grown in India, *C. citratus* is most widely cultivated in Central and South America [3].

Asian and Caribbean cuisines frequently use lemongrass, which has gained popularity around the world in recent decades. It is most valuable to industry as a fragrance in cosmetics (soaps, perfumes). However, lemongrass has been utilized in Ayurvedic herbalism as well as Asian folk medicine for its purported effects on the central nervous, respiratory and digestive systems as well as its antipyretic effect and beneficial effect in skin disorders [3]. The presence of essential oils in lemongrass is primarily responsible for its health benefits [8].

*C. citratus* is widely utilized in the folk medicine in several countries [8,9]. In Singapore, it is used to aid in digestion, to ease cold and flu symptoms, to treat insect bites [10]. In Africa, this species is used to treat cough, headache, athlete’s foot and cuts, whereas in India to treat fever, dysentery, stomachache, headache, insect bites, and rheumatism [11,12]. Some studies have described its analgesic, antipyretic, and anti-cancer effects in preclinical studies [13,14,15]. The essential oil and other extracts obtained from *C. citratus* have demonstrated anti-inflammatory, antimalaria, and antimutagenic activities [16,17]. The *C. citratus* essential oil exerted antimicrobial activities as well against a series of pathogenic bacteria, e.g., *Escherichia coli*, *Staphylococcus aureus*, *Bacillus cereus* and fungi, e.g., *Candida tropicalis*, in several studies [8,18,19,20,21,22,23].

In Asia, *C. flexuosus* is used as both a herbal beverage and a spice [24]. This plant has less important role in folk medicine. In certain regions of India, the leaves are used to reduce pain and fever [12] or cough and cold [25]. Its essential oil exhibited anti-inflammatory effect in vitro [26], was effective against several pathogen *E. coli* O157:H7 strains [27] and against biofilm formation of *Candida albicans* and *S. aureus* [28]. The *C. flexuosus* essential oil was reported to inhibit the proliferation of various cancer cells in vitro [29].

*C. pendulus* is used primarily in perfumery. In certain chemotypes, the elemicin content is very high in the essential oil (>50%), and hence these oils can be used as starting material for the synthesis of the antimalarial drug trimethoprim [30].

The primary components of LGEO, neral and geranial, are chemically different substances (Figure 1), yet they are typically referred to as citral in the studies describing their bioactivities. Citral, which is found in lemongrass oil, is widely responsible for the plant’s antibacterial properties [31]. The antifungal potential of the oil is also attributed to this compound [32]. Citral has been shown to be an effective antioxidant both in vivo and in vitro [33]. This substance has also been demonstrated to have antiproliferative properties against a variety of cancer cell types [34,35,36].

The use of LGEO is considered to be safe in accordance with the opinion of the Food and Drug Administration of USA, which qualified lemongrass oil (obtained from *C. citratus* and *C. flexuosus*) as generally recognized as safe (GRAS) as food additive [37]. *C. citratus* essential oil is not official in the European Pharmacopoeia, in contrary to the oil of *C. winterianus* obtained by steam distillation [38]. As fragrance ingredient, citral is not genotoxic, it is not expected to be phototoxic/photoallergenic, showed no local respiratory toxicity and was found not to be persistent and bioaccumulative [39].

The potential clinical applications of LGEO are diverse [32]. Despite the promising findings from some studies [32,40], the clinical application of LGEO in patients remains a topic of debate. Some experts have raised concerns about the safety and efficacy of using the oil in clinical practice, particularly when it is ingested or applied topically.

Given the current interest in the clinical application of LGEO, there is a need for a comprehensive review of the existing literature on this topic. The present paper presents a scoping review exploring and summarizing the existing literature on the clinical applications of LGEO to provide an overview of the potential therapeutic benefits. By synthesizing the existing evidence, this review can provide insights for healthcare professionals and researchers interested in exploring the clinical potential of LGEO in patient care.

## 2. Results

**Figure 2:**
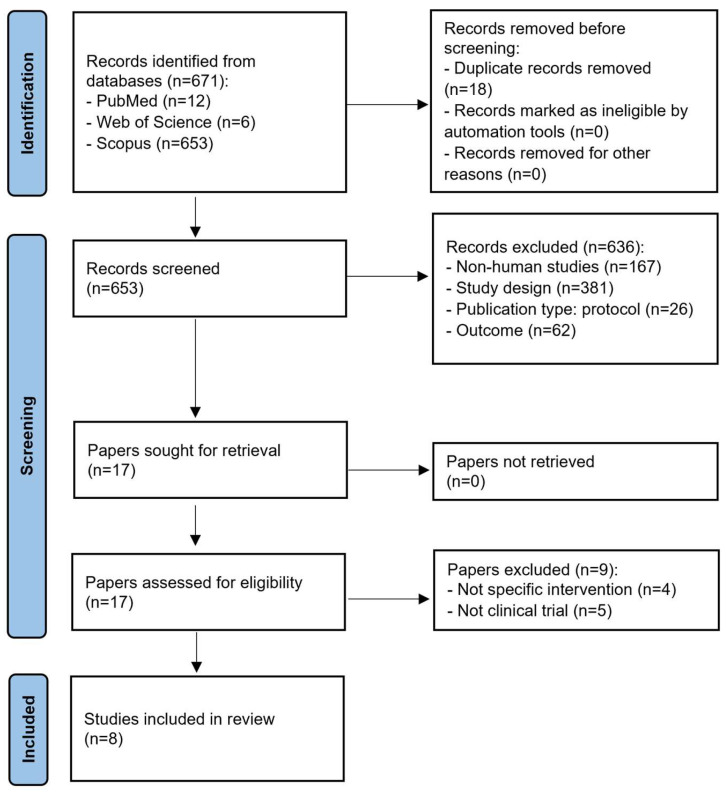
PRISMA flowchart diagram of article selection for a scoping review of the clinical use of lemongrass essential oil.

The search strategy employed a combination of keywords, including “lemongrass” “essential oil” and “clinical trial” (Supplementary Information S1). As a result of this search, a total of 671 records were identified from databases, including PubMed (*n* = 12), Web of Science (*n* = 6), and Scopus (*n* = 653) (Figure 2). After removing duplicates, 653 articles were assessed based on established inclusion and exclusion criteria. A total of 636 articles were excluded from the review that did not meet the established criteria. These exclusions were mainly due to being the population (i.e., non-human studies), study design, publication type, or outcome. Out of eight eligible articles, three studies did not provide detailed information about the randomization method, and two did not mention whether the treatment allocation to the patients was concealed. Furthermore, two studies exhibited performance bias, and six showed detection bias. The risk of bias summary and graph can be found in Supplementary Information S2.

Afterwards, the remaining 17 articles were subjected to a second screening performed through a face-to-face discussion and full-text analysis. After the second screening, we excluded nine articles that did not meet the established criteria (Figure 2). In summary, this scoping review is based on eight studies that met the inclusion criteria, providing valuable insights into the application of *C. citratus* and *C. flexuosus* essential oils in clinical aspects [41,42,43,44,45,46,47,48]. Based on the results of these studies published from 2013 to 2022, there have been evidences for the potential clinical applications of both the *C. citratus* and *C. flexuosus* oils in various healthcare areas.

Certain studies suggest that the *C. citratus* oil could be an effective alternative to chlorhexidine mouthwash for reducing plaque, gingivitis, and oral malodor (Table 1) [41,42,43]. Moreover, in patients with moderate chronic periodontitis, the adjunctive use of a mouthrinse containing *C. citratus* or *C. flexuosus* oil following scaling and root planing had a positive effect on clinical variables and on bacterial levels in the subgingival biofilm (Table 1) [44,45].

Furthermore, the results of a study indicate that *C. citratus* oil has potential as a natural antioxidant to be used as part of cosmetic products designed to prevent or treat signs of skin aging (Table 1) [46]. In addition, the *C. citratus* oil and the *C. flexuosus* oil demonstrated therapeutic potential for the treatment of pityriasis versicolor [47] and dandruff [48], respectively (Table 1).

## 3. Discussion

To the best our knowledge, this is the first scoping review that comprehensively surveyed the use of LGEO in clinical studies, therefore this review highlights the potential benefits of LGEO in oral care, skin care and hair care products. Our findings resonate with the increasing interest in herbal remedies for oral health, as highlighted in a recent study by Budala et al. [49]. This study underscores the significance of natural compounds in modern dental care, addressing the impact of microbial flora imbalance and showcasing the potential of herbal, animal, and microbial natural products in preventing and treating dental diseases [49]. This broader perspective enhances our discussion, offering a comparative assessment of LGEO’s effectiveness in oral care.

### 3.1. Oromucosal Application

Most of the trials focused on the effect of *C. citratus* and *C. flexuosus* oils on the oral mucosa [41,42,43,44,45]. These oils were applied as gel [45], mouthrinse [41,42,43,44], efficacy was compared to doxycycline gel [45], placebo [41,44] or chlorhexidine [42,43]. The concentration of the oils in the oromucosal gel was 2% [45], whereas the mouthrinses contained 0.25% [42,43], 0.5% [44] or 1% [41] oil. However, it should be noted that in one case the mouthrinse contained other essential oils as well (0.5% *Thymus zygis* essential oil and 0.5% *Rosmarinus officinalis* essential oil) [44]. In case of this combination product, the lemongrass oil was obtained from *C. flexuosus* [44], a related species of *C. citratus*. In one of the studies, it was not clear whether *C. citratus* or *C. flexuosus* oil was used [42]. The composition of the oil used in the applied products was not disclosed [42,43,44,45], except one study, where the main components of the essential oil were β-citral (neral, 34%) and α-citral (geranial, 45%) [41]. This composition corresponds to the chemical characteristics of *C. citratus* oil reported in the literature [8].

In case of oromucosal use, the efficacy was assessed in healthy volunteers [41,43], patients suffering in gingivitis [42] and periodontitis [44,45]. The use of different scoring systems makes it difficult to compare results even for similar groups of patients.

#### 3.1.1. Efficacy in Periodontitis

This scoping review suggests that the use of LGEO may be effective in the treatment of periodontitis. The mouthrinse containing *C. flexuosus* oil was superior to placebo [44] and the *C. citratus* oil had similar efficacy to that of doxycycline [45]. The adjunctive use of a mouthrinse containing essential oils, including *C. flexuosus* oil, following scaling and root planing (SRP) had a positive effect on clinical variables and on bacterial levels in the subgingival biofilm [44]. The test group showed improvements in attachment level (AL) and bleeding on probing (BOP) after three and/or six months compared to control group [44]. This might be due to the preventive potential of lemongrass oil against the microbial recolonization of periodontal pockets [50]. One study also supported that a locally administered 2% lemongrass (*C. citratus*) gel might be used as an adjunct to scaling and root planing in the treatment of chronic periodontitis [45]. Both the 2% lemongrass gel and 10% doxycycline gel groups had a significant reduction in mean scores of gingival index (GI), probing pocket depth (PPD), and clinical attachment level (CAL) clinical indices from baseline to the 1st and 3rd month follow-ups [45]. These results suggests that both the *C. citratus* and *C. flexuosus* oils might be effective as an adjunct to scaling and root planning in periodontitis. However, the presence of two additional essential oils that may also have therapeutic effects interferes with the assessment of effectiveness of the *C. flexuosus* oil in periodontitis [44].

#### 3.1.2. Efficacy in Gingivitis

One study indicated that lemongrass mouthwash could be an alternative of chlorhexidine in gingivitis [42]. This finding is corroborated by prior research that found LGEO to be beneficial in the treatment of gingivitis [50,51]. The inflammatory infiltration in gingivitis is characterized by the presence of lymphocytes, plasma cells, and neutrophils, which can disrupt the oxidative stress and antioxidant balance of the gingival tissues [51]. Natural antioxidants are crucial for maintaining tissue homeostasis in this condition. LGEO, with its abundance of natural antioxidants, may help to counteract oxidative stress and support healthy gingival tissues [52]. Moreover, the anti-inflammatory and antimicrobial properties of LGEO can effectively prevent microbial recolonization of periodontal pockets, promoting clinical resolution of gingival inflammation. Furthermore, its antioxidant activity can prevent periodontal tissue destruction and enhance healing [50].

#### 3.1.3. Prophylactic Use

In healthy children, a mouthwash containing *C. citratus* oil was effective in reducing plaque index (PI) and GI scores at both 14th and 21st day [43]. In healthy adults, the use of a *C. citratus* oil mouthrinse decreased malodour [41]. Moreover, a latest finding also demonstrated a significant reduction in both PI and GI values among patients undergoing fixed orthodontic treatment within a 21-day period when utilizing a 0.25% *C. citratus* oil mouthwash in comparison to the administration of chlorhexidine and oral prophylaxis [53]. The antimicrobial activity against different bacteria was demonstrated by different authors [41,44,45], and this might be related to the therapeutic efficacy. These finding are consistent with the results of Gao et al. [28], who reported that LGEO could reduce the viability of pathogenic microorganisms due to the citral content of the oil. The anti-biofilm activity of LGEO is likely due to its constituents including citral, limonene, citronellal, β-myrcene, linalool, and geraniol [54]. These terpenes are able to disrupt the lipid bilayer of cell membranes by penetrating between fatty acyl chains, leading to changes in membrane fluidity, surface alterations, and morphological modifications [55]. Additionally, they can reduce the adherence capacity of oral pathogens. As adherence is a crucial step in biofilm formation, these agents may have potential for preventing biofilm-associated infections [56].

### 3.2. Dermal Application

Along with the evaluation of lemongrass’ oromucosal effectiveness, its clinical effectiveness following dermal application has also been studied. LGEO exhibits antibacterial, antioxidant, and anti-inflammatory properties, making it a promising candidate for skincare applications [16,17,22,57]. The main components of LGEO, i.e., *trans*-citral and neral (*cis*-citral), have been shown to possess anti-inflammatory properties [58]. The *C. citratus* oil, when applied topically, had good prospects for clinical application for the treatment of pityriasis versicolor due to its safety and biological effects observed in vivo by mycological cure [47]. Although it was less effective than ketoconazole, its clinical efficacy was demonstrated [47]. The action mechanism of essential oils against microorganisms involves interference in the phospholipid bilayer of cell membrane, disruption of enzymatic systems, compromising of the bacteria genetic material, and formation of fatty acid hydroxyperoxidase [59].

Through its antioxidant activity, LGEO protects the skin against free radicals and mitigates the formation of wrinkles, photoaging, elastosis, dryness, and pigmentation processes [60]. Leelapornpisid et al. [46] used an essential oil blend (EOB) in which *C. citratus* oil was one of the components. Significant reduction of skin roughness was observed compared with untreated skin and placebo treatment. However, the contribution of *C. citratus* oil in this effect could not be objectively assessed due to the presence of other essential oils (*Zingiber officinale*, *Amomum uliginosum*, *Ocimum sanctum* and *Cananga odorata*). Furthermore, a clear protective effect against skin inflammation was attributed to *C. citratus* oil in a recent study using the methyl nicotinate microinflammation skin model [61]. LGEO may act as free radical scavenger, preventing oxidative damage of cellular components, and it also inhibits enzymes such as collagenase, elastase, and hyaluronidase. These enzymes are involved in the breakdown of important skin structures, and inhibition keeps the skin hydrated and elastic [62]. Moreover, LGEO can modulate the NF-κB pathway. By suppressing NF-κB, pro-inflammatory cytokine expression can be decreased, which can lead to more balanced and less inflammatory skin and anti-aging benefits [63].

The anti-inflammatory effects of LGEO can be explained due to the involvement of various molecules that partially hinder the release of substances that mediate inflammation [16]. Moreover, citral, a predominant element in LGEO, impedes the generation of IL-1b and IL-6 in alveolar macrophages stimulated with lipopolysaccharide (LPS) [16]. Essential oils containing citral, geranial, neral, and carvone as primary constituents have been observed to restrain the production of pro-inflammatory cytokines such as tumor necrosis factor alpha (TNF-α) [16].

### 3.3. Anti-Dandruff Effect

Evaluation of the effects of an anti-dandruff hair tonic containing *C. flexuosus* oil was performed by Chaisripipat et al. [48]. The application of hair tonics with 5, 10, or 15% oil reduced dandruff significantly at day 7th and increased the effect even more at day 14th. The hair tonic formulation with 10% of oil seemed to be the most effective preparation. LGEO has been shown to have antifungal properties, specifically against dandruff-associated pathogens [64,65]. LGEO’s antifungal effect is considered to be caused by disrupting the fungal cell membrane, altering the permeability of the membrane, and ultimately destroying the fungal cells. Furthermore, the antifungal effect of citral was linked to its interaction with membrane ergosterol, resulting in membrane instability and abnormality in cell mitochondria [66].

### 3.4. Safety

The LGEO is considered as a safe additive in many industrial areas, including food and feed processing and cosmetics production [6,67]. Toxicity investigations have indicated its safety in oral and dermal applications at low concentrations, however, the potential to cause mild to moderate adverse effects, mainly skin irritation, was also described [68,69,70]. By using the trypan blue dye exclusion test, LGEO demonstrated low toxicity at low concentrations; in contrast, in the methyl tetrazolium (MTT) assay it did not significantly reduce cell viability [70]. Other studies, however, have drawn attention to LGEO’s toxicity, particularly with regard to cancer cells. Citral has been shown to have anti-cancer properties by causing apoptosis in breast cancer cells [71].

### 3.5. Limitations and Perspectives

The encouraging findings of our review should be assessed while also taking limitations into consideration. The limitations include the small sample size and the lack of a placebo group in some of the studies reviewed. Additionally, the studies included in this review vary in terms of methodology, study population and outcome measures, which may limit the generalizability of the findings. In our findings, we acknowledge the limited number of studies available on LGEO’s therapeutic applications. While the evidence is promising, the scoping review underscores the need for further research to strengthen the robustness of the conclusions.

Future studies may advance our understanding of LGEO’s clinical applications. Well-designed controlled clinical trials with larger sample sizes, exploration of the mechanisms of action, optimization of dosing regimens, comprehensive safety assessment, investigation into bioavailability and pharmacokinetics, consideration of combination therapies, health economics studies are needed in order to better understand the role of LGEO in evidence-based medicine.

## 4. Materials and Methods

### 4.1. Study Design

A systematic literature search was conducted to identify eligible scientific papers published between 1 January 2013, and 1 November 2022. This scoping review was reported according to the Preferred Reporting Items for Systematic reviews and Meta-Analyses extension for Scoping Reviews (PRISMA-ScR) guidelines (Supplementary Information S3) [72]. All curated studies were then imported into the automated citation checking service Zotero (https://www.zotero.org/, accessed on 14 November 2023) to identify and remove duplicate records.

### 4.2. Search Strategy

The systematic literature search was performed using PubMed, Scopus and Web of Science databases on 1 November 2022. The search key included a combination of keywords “lemongrass” and “essential oil” and “clinical trial”. The search strategy for each database is described in the Supplementary material (Supplementary Information S1). The study population were adults, the intervention was LGEO, the comparator was any other treatment, including placebo or active control, the outcome was LGEO effect in clinical application.

### 4.3. Eligibility Criteria

Studies documenting the clinical application of LGEO in patients were included. Studies were eligible if they were published in English, peer-reviewed, and included human participants of any age, gender, or health status. Studies other than human clinical trials (e.g., review, animal study, in vitro, in vivo), focusing solely on the chemical composition or pharmacological properties of LGEO, and being not available in full text were excluded from the analysis.

### 4.4. Study Selection

Two independent reviewers (IYK, MIP) screened the titles and abstracts of all identified articles for eligibility using the predefined inclusion and exclusion criteria using Rayyan AI software (https://www.rayyan.ai/, accessed on 14 November 2023) [73]. The data extraction screening included information on study design, participant characteristics, intervention details, outcomes, and limitations. Any disagreements were resolved through discussion and consensus. The full text of potentially eligible articles was then reviewed by the same two reviewers (IYK, MIP) using the same criteria. A third round of screening was also conducted to identify additional studies that met the inclusion criteria based on the full text review of the reference lists. Two reviewers (IYK, MIP) separately extracted data, then cross-checked and compiled it by IYK. The synthesis involved a descriptive summary of the study characteristics and a thematic analysis of the reported outcomes in Microsoft Office Excel 2019 using a standardized template. The data included the author, year, country, population/participants, study design, intervention, duration of intervention, evaluation, and outcome provided (see Table 1).

### 4.5. Study Risk of Bias Assessment

The included studies were assessed for methodological quality using the Cochrane risk-of-bias tool by RevMan software version 5.4.1. IYK and MIP independently conducted the appraisal of study quality. The disagreements between the two reviewers were resolved through discussion. In case no consensus was reached, DC, CV, and MT were consulted as additional reviewers.

## 5. Conclusions

LGEO has shown potential for use in various therapeutic applications, including as a mouthwash for reducing plaque, improving gingivitis, and as an adjunct to scaling and root planing in chronic periodontitis patients. LGEO has also demonstrated promising results in the treatment of skin diseases related to infections and as cosmetic product for the prevention or treatment of signs of skin aging. These findings suggest that oromucosally or dermally applied LGEO could be a valuable complementary or alternative tool in the therapy of different diseases. While the scoping review has provided valuable information on the potential therapeutic applications of LGEO, it is important to note that the evidence is still limited, and further research is necessary to fully evaluate its effectiveness and safety. Furthermore, studies are required to explore the potential mechanisms of action and interactions with other medications, as well as the safety and tolerability of long-term use in various populations.

## Figures and Tables

**Table 1:** Main characteristics of the studies published on the clinical applications of lemongrass essential oil (LGEO) between 1 January 2013, and 1 November 2022.

Author (Year)	Country and Participants	Study Design	Intervention	Duration of Intervention	Evaluation	Outcome
Satthanakul et al. [41] (2015)	Thailand, 20 healthy volunteers	Double-blinded, randomized, placebo-controlled clinical trial	Group A (*n* = 10): LGEO (*Cymbopogon citratus*) mouthrinse 2×/day in the morning and night. Group B (*n* = 10): placebo mouthrinse 2×/day in the morning and night.	7 days	The concentration of volatile sulphur compounds in breath measured by halimeter. Organoleptic test using a 9-point hedonic scale.	LGEO reduced concentration of volatile sulphur compounds in breath significantly in comparison with placebo both after 1-min once rinse and 7-days treatment. Participants were more satisfied with the LGEO rinse (overall satisfaction taste, breath freshness).
Dany et al. [42] (2015)	India, 60 patients with mild to moderate gingivitis	Double-blinded, randomized, controlled clinical trial	Group A (*n* = 20): 0.25% LGEO (*C. citratus* or *Cymbopogon flexuosus*) mouthwash 2× daily + toothbrushing. Group B (*n* = 20): 0.2% chlorhexidine mouthwash 2× daily + toothbrushing. Group C (*n* = 20): toothbrushing only.	21 days (follow-up at 14 and 21 days)	Plaque index (PI) and gingival index (GI) scores	PI and GI improved significantly after 14 and 21 days in all groups. A greater reduction in PI and GI score was recorded in the LGEO group after 14 and 21 days, followed by chlorhexidine mouthwash group, followed by oral prophylaxis only group.
Akula et al. [43] (2021)	India, 60 healthy children between 9–12 years	Single-blinded, randomized controlled trial	Group A (*n* = 20): 0.25% LGEO (*C. citratus*) mouthwash 2× daily. Group B (*n* = 20): 0.2% chlorhexidine mouthwash 2× daily. Group C (*n* = 20): oral prophylaxis alone, *n* = 20).	21 days (follow-up after 14 and 21 days)	Plaque pH, plaque index (PI), and gingival index (GI)	Intragroup comparison of PI and GI showed a significant decrease between 14 and 21 days in groups A and B, whereas mean plaque pH increased only in group A at day 21 compared with baseline. Intergroup comparison of the PI scores of the three groups at days 1, 14, and 21 did not show any statistically significant differences, but the mean GI was significantly different among the three groups at days 14 and 21.
Azad et al. [44] (2016)	Germany, 46 patients with moderate chronic periodontitis undergoing scaling and root planing	Double-blinded, randomized, placebo-controlled clinical trial	Group A (*n* = 23): LGEO (*C. flexuosus*) mouthrinse 2× daily. Group B (*n* = 23): placebo mouthrinse 2× daily.	14 days (follow up after 3 and 6 months)	Probing depth (PD), attachment level (AL), bleeding on probing (BOP) and modified sulcus bleeding index (SBI) were measured at baseline, 3 months, and 6 months, and subgingival plaque was assessed for periodontitis-associated bacteria.	AL, PD, BOP, and SBI improved significantly in both groups after 3 and 6 months. AL improved significantly better in the group A after 3 and 6 months. The improvement of BOP was also better in the group A after three months. There was no significant difference between the groups at SBI. Test group had more reduction in *Treponema denticola* and *Fusobacterium nucleatum* after 3 months and *Tannerella forsythia* after 6 months. *Prevotella micra* and *Campylobacter rectus* decreased significantly in both groups after 3 months.
Mittal et al. [45] (2022)	India, 40 subjects suffering from chronic periodontitis	Double-blinded, randomized controlled trial	Group A (*n* = 20): 2% LGEO (*C. citratus*) gel administered into the periodontal pocket after scaling and root planning. Group B (*n* = 20): 10% doxycycline hyclate gel administered into the periodontal pocket after scaling and root planning. Both groups were advised to use 0.2% chlorhexidine mouthrinse.	3 months (follow-up after 1 and 3 months)	The clinical assessments of gingival index (GI), plaque index (PI), probing pocket depth (PPD), and clinical attachment level (CAL); colony forming unit (CFU) scores of *Porphyromonas gingivalis*, *Actinomyces naeslundii*, and *Prevotella intermedia*	Both the 2% LGEO gel and 10% doxycycline gel significantly improved clinical mean scores after 1 and 3 months (except PI in the LGEO group) and reduced CFU scores for periodontal pathogens.
Leelapornpisid et al. [46] (2015)	Thailand, 29 healthy volunteers	Double-blinded, placebo-controlled clinical trial	All participants used an LGEO (*C. citratus*) containing body cream and a placebo cream twice daily on the forearm.	4 weeks	Skin condition was evaluated using the Skin Visiometer and skin moisture was evaluated with the Corneometer^®^ at three test sites (untreated, active-cream, and placebo-cream).	LGEO cream significantly improved surface texture compared to baseline, whereas in case of untreated and placebo-treated surfaces no such effects were observed. Both LGEO cream and placebo improved skin hydration.
Carmo et al. [47] (2013)	Brazil, 96 patients diagnosed with pityriasis versicolor	Safety (I) phase: open clinical trial. Efficacy (II) phase: randomized, open clinical trial.	Phase I: 20 patients used an LGEO (*C. citratus*) shampoo 3× weekly and an LGEO cream 2× daily. Phase II: Group A (*n* = 30): LGEO shampoo 3× weekly and an LGEO cream 2× daily. Group B (*n* = 30): ketoconazole shampoo 3× weekly and a ketoconazole cream 2× daily.	40 days (each phase)	Phase I: adverse reactions Phase II: rate of mycological cure	Phase I showed no adverse events (except one case of burning sensation on the scalp after applying the shampoo). Phase II, LGEO had a 60% mycological cure rate, while ketoconazole had over 80%. Both treatments were effective, however, ketoconazole was significantly more effective.
Chaisripipat et al. [48] (2015)	Thailand, 30 healthy volunteers experiencing dandruff	Double-blinded, randomized, placebo-controlled trial	Group A (*n* = 10): application of 5 drops of a head tonic containing 5% LGEO (*C. flexuosus*) in one side of the head and a placebo hair tonic on the other side of the head, 2× daily. Group B (*n* = 10): application of 5 drops of a head tonic containing 10% LGEO in one side of the head and a placebo hair tonic on the other side of the head, 2× daily. Group C (*n* = 10): application of 5 drops of a head tonic containing 15% LGEO in one side of the head and a placebo hair tonic on the other side of the head, 2× daily.	14 days (follow-up: 7 and 14 days)	Reduction of dandruff using the D-Squame^®^ scale	The application of LGEO hair tonics with 5, 10, or 15% reduced dandruff significantly (*p* < 0.005) at day 7 (33, 75, and 51%) and increased the effect even more (*p* < 0.005) at day 14 (52, 81, and 74%). Placebo treatment was less effective, but also showed significant efficacy in groups B and C after 21 days.

## Data Availability

Data is contained within the article.

## Supplementary Materials

The following supporting information can be downloaded at: https://www.mdpi.com/article/10.3390/ph17020159/s1, Supplementary Information S1: Description of search strategies applied for PubMed, Scopus and Web of Science databases; Supplementary Information S2: Risk of bias assessment results obtained using RevMan software version 5.4.1. (A) Summary of risk of bias analysis performed for each included study [41,42,43,44,45,46,47,48]. (B) Graph on each risk of bias item presented as percentages across all included studies. Low risk of bias, green; unclear risk of bias, yellow; high risk of bias, red; Supplementary Information S3: Preferred Reporting Items for Systematic reviews and Meta-Analyses extension for Scoping Reviews (PRISMA-ScR) checklist.
